# Nonparametric estimation of median survival times with applications to multi-site or multi-center studies

**DOI:** 10.1371/journal.pone.0197295

**Published:** 2018-05-17

**Authors:** Mohammad H. Rahbar, Sangbum Choi, Chuan Hong, Liang Zhu, Sangchoon Jeon, Joseph C. Gardiner

**Affiliations:** 1 Department of Epidemiology, Human Genetics, and Environmental Sciences, University of Texas School of Public Health at Houston, Houston, TX 77030, United States of America; 2 Division of Clinical and Translational Sciences, Department of Internal Medicine, McGovern Medical School; University of Texas Health Science Center at Houston, Houston, TX 77030, United States of America; 3 Biostatistics/Epidemiology/Research Design component, Center for Clinical and Translational Sciences, University of Texas Health Science Center at Houston, Houston, TX 77030, United States of America; 4 Department of Statistics, Korea University, Seoul, South Korea; 5 Department of Biostatistics, Harvard University, Boston, MA 02115, United States of America; 6 Division of Acute Care/Health Systems, School of Nursing, Yale University, West Haven, CT 27399, United States of America; 7 Department of Epidemiology and Biostatistics, Michigan State University, Lansing, MI 48824, United States of America; University of Georgia, UNITED STATES

## Abstract

We propose a nonparametric shrinkage estimator for the median survival times from several independent samples of right-censored data, which combines the samples and hypothesis information to improve the efficiency. We compare efficiency of the proposed shrinkage estimation procedure to unrestricted estimator and combined estimator through extensive simulation studies. Our results indicate that performance of these estimators depends on the strength of homogeneity of the medians. When homogeneity holds, the combined estimator is the most efficient estimator. However, it becomes inconsistent when homogeneity fails. On the other hand, the proposed shrinkage estimator remains efficient. Its efficiency decreases as the equality of the survival medians is deviated, but is expected to be as good as or equal to the unrestricted estimator. Our simulation studies also indicate that the proposed shrinkage estimator is robust to moderate levels of censoring. We demonstrate application of these methods to estimating median time for trauma patients to receive red blood cells in the Prospective Observational Multi-center Major Trauma Transfusion (PROMMTT) study.

## Introduction

Multi-site and multi-center studies have become very popular in clinical and translational sciences in the past two decades. Although multi-site studies allow for increased enrollment rate and improved generalizability to the target population [1], they introduce additional statistical challenges in the study design and analysis of data from these studies. For example, multi-site studies could lead to non-homogeneous sub-samples due to the differences in the study sites. This is particularly relevant to trauma and emergency care research.

Having served as the Data Coordinating Center for the Prospective Observational Multicenter Major Trauma Transfusion (PROMMTT) study [1], we have identified a number of challenges in analysis of time to event data from PROMMTT. For example, in blood transfusion research, time to receive the first unit of red blood cells (RBCs) is one of important surrogates to measure how rapidly trauma patients receive blood transfusion. However, such data were often collected from multi-sites. Due to the fact that trauma centers may have their own guideline and practice to manage trauma patients, different sites may not only contribute different number of patients but also have different distributions of time to event of interest with different levels of censoring and variability. As a result, analysis of data from multi-site studies requires an exploratory step prior to pooling samples from different sites.

In some medical research, particularly in multi-site randomized clinical trials (e.g., for trauma/blood transfusion), when the main outcome of interest is time to observe a certain outcome of interest (e.g., time to receive the first unit of RBCs), complete observations in all patients are not usually available due to death before receiving a fixed number of units of RBCs, which results in right-censored data. [2] introduced nonparametric estimation of the survival curve, based on right-censored data. Nonparametric estimation of the mean survival time has been studied by many investigators including [3, 4] and [5, 6], and [7] have extensive discussions on nonparametric estimation of the mean and quantiles of the survival function. [8] introduced a nonparametric procedure for testing the equality of median survival times from k-independent samples of right censored data. [9] introduced an alternative to the BC test, which may perform better than the BC test under certain situations. To deal with the inflated Type I error rates when sample sizes are small, more recently, [10] extended Mood’s median test for uncensored data to the setting of survival data.

The main aim of this research is to propose an improved nonparametric method for estimation of the median survival time from right-censored data from k-independent samples when there is uncertainty regarding the homogeneity of the k-population medians. We compare the proposed estimator to other two commonly used estimators asymptotically, and through extensive simulations. In addition, we demonstrate application of this method to data from the PROMMTT study ([11, 12]).

The remainder of the article is organized as follows. In Section 2, we propose the estimation strategies for median survival times and make comparisons among different estimators. In Section 3, we present the results from our simulation study comparing the performance of the proposed estimator against the other two commonly used estimators based on mean square errors (MSE). In Section 4, we demonstrate an application of our proposed method to data from PROMMTT ([1, 11]). Section 5 is devoted to concluding remarks with some discussion.

## Materials and methods

We consider the nonparametric estimation of median survival times based on right-censored data in the presence of uncertain prior information in a *k*-independent sample situation. We assume here that $T_{i1},\cdots ,T_{in_{i}},(i=1,\cdots ,k)$ are random samples selected from *k* populations with *n*_*i*_ observations taken from the *i*-th population. We will refer to *T*_*ij*_ as to the survival time of *j*-th subject in the *i*-th population. Due to censoring at time *C*_*ij*_, the survival time or time-to-event may not be observable in some subjects. Therefore, for each subject, the data are recorded in the form (*Y*_*ij*_, *δ*_*ij*_), *j* = 1, ⋯, *n*_*i*_, *i* = 1, ⋯, *k*, where *Y*_*ij*_ = min(*T*_*ij*_, *C*_*ij*_), *δ*_*ij*_ = *I*(*T*_*ij*_ ≤ *C*_*ij*_), and *I*(⋅) is the indicator function. We assume that random variables *T*_*ij*_ and *C*_*ij*_ are independent with continuous survival distributions *F*_*i*_(*x*) = *P*(*T*_*ij*_ > *x*) and *G*_*i*_(*y*) = *P*(*C*_*ij*_ > *y*), respectively.

### 0.1 Unrestricted estimator (UE)

A straightforward way to estimate the median-parameter vector can be defined as,

$$\begin{array}{c} {\hat{\Theta }}^{UE}=\hat{\Theta }={\left({\hat{\theta }}_{1},\cdots ,{\hat{\theta }}_{k}\right)}^{T}\;. \end{array}$$ (2.1)

where ${\hat{\theta }}_{i}={\hat{F}}_{i}^{-1}\left(0.5\right)$ and ${\hat{F}}_{i}\left(p\right)=\inf\left\{t:{\hat{F}}_{i}\left(t\right)\geq p\right\}$ for *i* = 1, ⋯, *k*.

We call this estimator as unrestricted estimator (UE) of Θ. This estimator is usually used when no hypothesis information is available on Θ. For example, in a multi-site study with *k* sites one can provide site-specific estimates for median survival time without combing the data from all sites. All the estimators for the *k* components of the vector are independent.

For each *i* = 1, ⋯, *k*, $\sqrt{n_{i}}\left\{{\hat{F}}_{i}^{-1}\left(p\right)-F_{i}^{-1}\left(p\right)\right\}$ converges in distribution to a normal random variable with mean 0 and variance $\Psi_{i}\left(p\right)=p\left(1-p\right)/\left\{f_{i}{\left(F_{i}^{-1}\left(p\right)\right)}^{2}\right\}$. Hence operationally, ${\hat{F}}_{i}^{-1}\left(p\right)$ is asymptotically normal with mean $F_{i}^{-1}\left(p\right)$ and variance Ψ_*i*_(*p*)/*n*_*i*_.

### 0.2 Combined estimator (CE)

Θ can also be estimated by combining the sample and hypothesis information under the assumption of homogeneity of the *k* medians given by

$$\begin{array}{c} H_{0}:\theta_{1}=\cdots =\theta_{k}=\theta_{0}\;\left(\text{unknown}\right). \end{array}$$ (2.2)

We can use this additional information together with the sample information to obtain improved estimators. Under the null hypothesis (2.2), we consider the combined/restricted estimator (CE) of Θ defined by

$$\begin{array}{c} {\hat{\Theta }}^{CE}={\left({\hat{\theta }}_{n}^{CE},\cdots ,{\hat{\theta }}_{n}^{CE}\right)}^{T},\;{\hat{\theta }}_{n}^{CE}=\sum_{i=1}^{k}\frac{n_{i}/{\hat{\sigma }}_{i}^{2}}{n_{1}/{\hat{\sigma }}_{1}^{2}+\cdots +n_{k}/{\hat{\sigma }}_{k}^{2}}{\hat{\theta }}_{i}\;. \end{array}$$ (2.3)

This estimator is expressed as a linear combination of the ${\hat{\theta }}_{i}’\text{s}$, i.e., ${\hat{\theta }}_{n}^{CE}=\sum_{i=1}^{k}{\hat{L}}_{i}{\hat{\theta }}_{i}$, where ${\hat{L}}_{i}=\left(n_{i}/{\hat{\sigma }}_{i}^{2}\right)/\left(n_{1}/{\hat{\sigma }}_{1}^{2}+\cdots +n_{k}/{\hat{\sigma }}_{k}^{2}\right)$, where ${\hat{\sigma }}_{i}^{2}$ can be estimated by $Var({\hat{F}}_{i}\left({\hat{\theta }}_{i}\right))/{\hat{f}}_{i}^{2}\left({\hat{\theta }}_{i}\right)$ for *i* = 1, ⋯, *k*. However, in order to avoid the difficulty in estimating the density function, we used the bootstrap to estimate the variance of the estimated median, following [9].

For the preliminary test on *H*_0_ in (2.2), we consider the following test statistic that is defined by the normalized distance of $\hat{\Theta }$ from ${\hat{\Theta }}^{CE}$:

$$\begin{array}{c} \Lambda_{n}=n\left(\hat{\Theta }-{\hat{\Theta }}^{CE}\right){\hat{\Gamma }}_{n}^{-1}\left(\hat{\Theta }-{\hat{\Theta }}^{CE}\right)\;, \end{array}$$ (2.4)

where

$$\begin{array}{c} {\hat{\Gamma }}_{n}=[\begin{array}{cccc} {\hat{\gamma }}_{n_{1}} & {\hat{\gamma }}_{n_{21}} & \cdots & {\hat{\gamma }}_{n_{k1}}\\ {\hat{\gamma }}_{n_{12}} & {\hat{\gamma }}_{n_{2}} & & {\hat{\gamma }}_{n_{k2}}\\ & \cdots & \cdots & \\ {\hat{\gamma }}_{n_{1k}} & {\hat{\gamma }}_{n_{2k}} & & {\hat{\gamma }}_{n_{k}} \end{array}]\;, \end{array}$$ (2.5)

where

$$\begin{array}{c} {\hat{\gamma }}_{n_{i}}={\hat{\sigma }}^{2}\omega_{i,n}^{-1}{\left(1-{\hat{L}}_{i}\right)}^{2}+\sum_{j\neq i}^{k}{\hat{\sigma }}_{j}^{2}\omega_{j,n}^{-1}{\hat{L}}_{j}^{2}\;, \end{array}$$

$$\begin{array}{c} {\hat{\gamma }}_{n_{ij}}=-{\hat{L}}_{i}\omega_{i,n}^{-1}{\hat{\sigma }}_{i}^{2}-{\hat{L}}_{j}\omega_{j,n}^{-1}{\hat{\sigma }}_{i}^{2}+\sum_{l=1}^{k}{\hat{\sigma }}_{l}^{2}\omega_{l,n}^{-1}{\hat{L}}_{l}^{2}\;,\omega_{i,n}=n_{i}/n,i,j=1,\cdots ,k. \end{array}$$

We assume that *ω*_*i*_ = lim_*n*→∞_ *ω*_*i*,*n*_ is fixed for *i* = 1, ⋯, *k*, and $\Gamma =\lim_{n\to \infty }{\hat{\Gamma }}_{n}$ exists and is nonsingular. It is shown that under the null hypothesis for large n, Λ_*n*_ follows the central *χ*^2^ distribution with *k* − 1 degrees of freedom [9]. For given *α*, the critical value of Λ_*n*_ may be approximated by $\chi_{k-1,\alpha }^{2}$, the upper 100*α*% point of the chi-square distribution with *k* − 1 degrees of freedom. More details can be found in [9].

### Positive-part shrinkage estimator (PP)

The combined estimator works well only when the null hypothesis (2.2) holds. If the null hypothesis (2.2) is rejected, we propose another estimator based on the James-Stein type shrinkage estimator (SSE) [12] which is defined by

$$\begin{array}{c} {\hat{\Theta }}^{JS}=\hat{\Theta }-\left\{\left(k-3\right)\Lambda_{n}^{-1}\right\}\left(\hat{\Theta }-{\hat{\Theta }}^{CE}\right),\;k\geq 4\;. \end{array}$$ (2.6)

The Stein-type estimator in (2.6) is not sensitive to departure from *H*_0_, and will provide uniform improvement in terms of efficiency for the entire parameter space of Θ. It is, however, not a convex combination of ${\hat{\Theta }}^{CE}$ and $\hat{\Theta }$. Also, this estimator may not remain nonnegative. To avoid this strange behavior, we truncate ${\hat{\Theta }}^{JS}$ at positivity boundary by adding an extra term to (2.6), which leads to a convex combination of $\hat{\Theta }$ and ${\hat{\Theta }}^{CE}$, namely, the positive-part shrinkage estimator (PP). When *k* ≥ 4, the positive-part shrinkage estimator is defined as follows:

$$\begin{array}{c} {\hat{\Theta }}^{PP}=\hat{\Theta }-\left(k-3\right)\Lambda_{n}^{-1}\left(\hat{\Theta }-{\hat{\Theta }}^{CE}\right)-\left\{1-\left(k-3\right)\Lambda_{n}^{-1}\right\}I\left(\Lambda_{n}<k-3\right)\left(\hat{\Theta }-{\hat{\Theta }}^{CE}\right)\;. \end{array}$$ (2.7)

where $\hat{\Theta }$, ${\hat{\Theta }}^{CE}$ and Λ_*n*_ were defined in Sections 2.1 and 2.2.

### 0.3 Comparison of ${\hat{\Theta }}^{UE}$, ${\hat{\Theta }}^{CE}$ and ${\hat{\Theta }}^{PP}$

In this Section, we compare the performance of ${\hat{\Theta }}^{UE}$, ${\hat{\Theta }}^{CE}$ and ${\hat{\Theta }}^{PP}$ by asymptotic distribution quadratic risk function [13]. For an estimator Θ*, define the weighted quadratic loss function of the form *L*(Θ*, Θ) = *n*(Θ* − Θ)^*T*^ *W*(Θ* − Θ), where *W* is a positive-definite matrix of weights. The expectation of the loss function *R*_0_(Θ*, Θ) = *E*[*L*(Θ*, Θ)] is called the risk function. The performance of the estimators can be evaluated by comparing the risk functions and an estimator with a smaller risk is preferred.

Since the test statistic in (2.2) is consistent for fixed Θ when Θ ∉ *H*_0_, ${\hat{\Theta }}^{PP}$ is asymptotically equivalent to ${\hat{\Theta }}^{UE}$ for fixed alternatives, this makes it difficult to compare their performance [14]. Alternatively, we may evaluate the asymptotic performance of each estimator under the following contiguous sequence of alternatives:

$$\begin{array}{c} K_{n}:\Theta =\Theta_{n},\;\Theta_{n}=\Theta_{0}+\varphi /\sqrt{n}\;, \end{array}$$ (2.8)

where *φ* is a fixed vector and Θ_0_ = (*θ*_0_, ⋯, *θ*_0_). The risk function *R*_0_(Θ*, Θ) = *E*[*L*(Θ*, Θ)] can be written as *R*_0_(Θ*, Θ) = *nE*[(Θ* − Θ)^*T*^ *W*(Θ* − Θ)] = *ntr*(*W*Γ*) where Γ* is the covariance matrix of Θ*. Then, considering the asymptotic distribution of $\sqrt{n}\left(\Theta^{*}-\Theta \right)$, we can define the asymptotic distribution quadratic risk (ADQR) as *R*(Θ*, Θ) = *tr*(*W*Γ) where Γ is the asymptotic covariance matirx. To facilitate the numerical computation and general discussion, we consider the particular case with *W* = Γ^ − 1^. Then, following similar arguments in [14], and define Δ = (*J*Ω − *I*_*k*_)Θ with $J=1_{k}1_{k}^{\prime }$ and $\Omega ={\sigma_{i}}^{2}I_{\left(k\times k\right)}$, and Δ* = Δ^*T*^ *W*Δ, we can demonstrate that

$$\begin{array}{c} R\left({\hat{\Theta }}^{UE},\Theta \right)=k\;, \end{array}$$ (2.9)

$$\begin{array}{c} R\left({\hat{\Theta }}^{CE},\Theta \right)=1+\Delta^{*}\;, \end{array}$$ (2.10)

$$\begin{array}{c} R\left({\hat{\Theta }}^{JS},\Theta \right)=k+\Delta^{*}\left(k-3\right)\left(k+1\right)E\left\{\chi_{k+3}^{-4}\left(\Delta \right)\right\}-\left(k-1\right)\left(k-3\right)\left[2E\left\{\chi_{k+1}^{-2}\left(\Delta \right)\right\}-\left(k-3\right)E\left\{\chi_{k+1}^{-4}\left(\Delta \right)\right\}\right]\;, \end{array}$$ (2.11)

and

$$\begin{array}{c} R\left({\hat{\Theta }}^{PP},\Theta \right)=R\left({\hat{\Theta }}^{JS},\Theta \right)-\left(k-1\right)E\left[\left\{1-\left(k-3\right)\chi_{k+1}^{-2}\left(\Delta \right)\right\}I\left\{\chi_{k+1}^{2}\left(\Delta \right)\leq k-3\right\}\right]\\ +\Delta^{*}\left[2E\left[\left\{1-\left(k-3\right)\chi_{k+1}^{-2}\left(\Delta \right)\right\}I\left\{\chi_{k+1}^{2}\left(\Delta \right)\leq k-3\right\}\right]\right]\\ -E[{\left\{1-\left(k-3\right)\chi_{k+3}^{-2}\left(\Delta \right)\right\}}^{2}I\left\{\chi_{k+3}^{2}\left(\Delta \right)\leq k-3\right\}]\;. \end{array}$$ (2.12)

An estimator Θ* is said to asymptotically dominate an estimator Θ^0^ if *R*(Θ*, Θ) ≤ *R*(Θ^0^, Θ), i.e., if the ADQR of Θ* is smaller for at least some value of Θ, and the ADQR does not exceed that of Θ^0^ for any value of Θ. Further, Θ* strictly dominates Θ^0^ if *R*(Θ*, Θ) < *R*(Θ^0^, Θ) for some (Θ, *W*). At Δ* = 0, that is, under the null, the dominance of the estimators is usually observed as ${\hat{\Theta }}^{CE}≻{\hat{\Theta }}^{UE}$, where the notation ≻ stands for dominance in terms of risk performance. For all Δ* and *k* ≥ 4, ${\hat{\Theta }}^{PP}≻{\hat{\Theta }}^{UE}$ is satisfied, that is, ${\hat{\Theta }}^{PP}$ asymptotically dominates ${\hat{\Theta }}^{UE}$ under local alternatives. Thus, we conclude that ${\hat{\Theta }}^{PP}$ consistently performs better than ${\hat{\Theta }}^{UE}$ in the entire parameter space induced by Δ*. The gain in risk over ${\hat{\Theta }}^{UE}$ is substantial when Δ* = 0 or near 0.

## Simulation studies

We conducted extensive simulation studies to examine the performance of the proposed estimators in situations with different degrees of departure from the assumption of homogeneity and censoring rates.

In order to evaluate the effect of the departure from the null hypothesis, we generated samples with median Θ_*ϵ*_ = (*θ*_0_ − 3*ϵ*, *θ*_0_ − *ϵ*, *θ*_0_ + *ϵ*, *θ*_0_ + 3*ϵ*) by varying *ϵ* ≥ 0 from uniform distributions on (*θ*_*i*_ − *a*_*i*_/*c*_*i*_, *θ*_*i*_ + *a*_*i*_/*c*_*i*_), where *a*_*i*_, *c*_*i*_ > 0. Let $d_{\epsilon }=\frac{1}{\sqrt{20}}\left|\right|\theta_{\epsilon }-\theta_{0}\left|\right|$, where ||⋅|| is the Euclidean norm. Various values of *ϵ* ∈ [0, 1] have been considered to achieve different *d*_*ϵ*_. The *k* samples of censoring variables {*Y*_1*i*_, ⋯, *Y*_*n*_*i*_*i*_} were generated from uniform distributions on (*θ*_*i*_ − *a*_*i*_/*c*_*i*_, *θ*_*i*_ + *a*_*i*_/*c*_*i*_ + *η*), where *η* is a fixed value to achieve a desired level of censoring (e.g., *p* = 0.3). We set Θ_0_ = (6, 6, 6, 6), *a* = (2, 2, 2, 2), and *c* = (1, 2, 1, 2). The simulation procedure was repeated 1000 times for *k* = 4 independent samples with size of 100 for each group.

**Fig 1:**
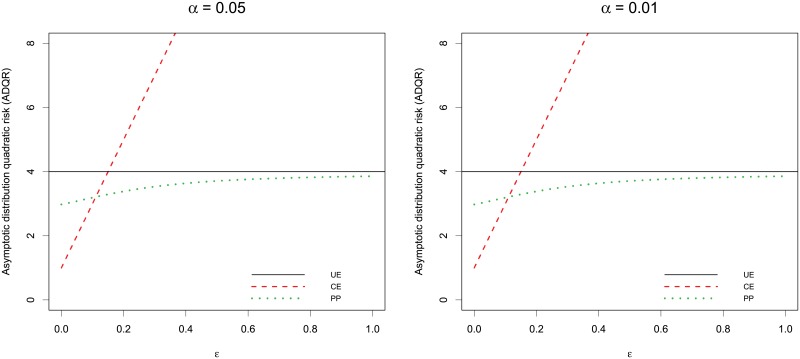
The asymptotic distributional quadratic risk (ADQR) performance of the estimators.

The simulation results of REs and the comparative plots are presented in Table 1 and Fig 1. The performance of Θ* was measured by the relative efficiency (RE), i.e., comparing its MSE with that of Θ^*UE*^, defined as $RE\left({\hat{\Theta }}^{UE},{\hat{\Theta }}^{*}\right)=MSE\left({\hat{\Theta }}^{UE}\right)/MSE\left({\hat{\Theta }}^{*}\right)$, where ${\hat{\Theta }}^{*}$ is one of the estimators (${\hat{\Theta }}^{CE}$ or ${\hat{\Theta }}^{PP}$) considered in this study. The amount by which a RE is larger than 1 indicates the degree of superiority of the estimator ${\hat{\Theta }}^{*}$ over ${\hat{\Theta }}^{UE}$. As highlighted in [9], to avoid difficulty in estimating the densitity function, we used boostrapping to estimate the variance of the estimated median. We also compare the performance of ${\hat{\Theta }}^{*}$ by asymptotic distribution quadratic risk described in Section 2.4.

**Table 1:** Simulated relative efficiency (RE) for combined (CE) and positive part shrinkage (PP) estimators relative to unrestricted estimator (UE) for different values of *ϵ*.

Distribution	Type	Relative efficiency (RE)					
		*ϵ* = 0	*ϵ* = 0.05	*ϵ* = 0.1	*ϵ* = 0.3	*ϵ* = 0.5	*ϵ* = 1.0
Uniform	CE	5.7087	1.7339	0.4185	0.0476	0.0181	0.0045
	PP	1.5629	1.3105	1.0358	1.0049	1.0073	1.0000

Table 1 shows that when *d*_*ϵ*_ = 0 or near zero, ${\hat{\Theta }}^{CE}$ outperforms all other estimators. However, as *d*_*ϵ*_ moves away from 0, the risk of ${\hat{\Theta }}^{CE}$ become unbounded, making it very inefficient. Overall, ${\hat{\Theta }}^{PP}$ maintain its superiority over other estimators for a wide range of *d*_*ϵ*_. This clearly suggests that ${\hat{\Theta }}^{PP}$ is preferred as there always remains uncertainty about level of heterogeneity between survival medians. In situations where the assumed model is grossly wrong, ${\hat{\Theta }}^{PP}$ is expected to be as good or equal to ${\hat{\Theta }}^{UE}$. The asymptotic behavior of ADQR over *d*_*ϵ*_ is illustrated in Fig 1 under different significance alpha levels. It shows that ${\hat{\Theta }}^{PP}$ may has smaller risks and thus be more efficient upon ${\hat{\Theta }}^{UE}$ under the null or near. The ADQR of ${\hat{\Theta }}^{CE}$ approaches infinity as *d*_*ϵ*_ grows. Overall, ${\hat{\Theta }}^{PP}$ has a better performance in the entire parameter space.

Next, we study scenarios with different distributions and censoring rates. In each scenario, 50 or 100 samples were generated from *k* = 4 subpopulations. The distributions considered in Tables 2 and 3 are (i) a uniform distribution with median Θ_0_ = (6,6,6.5,6), (ii) a log-normal distribution with Θ_0_ = (403.43, 665.14, 403.43, 665.14), and (iii) an exponential distribution with Θ_0_ = (6.69, 6.58, 7.0, 6.43). The distributions considered in Tables 4 and 5 are mixed distributions with (iv) two subpopulations had a uniform distribution and the other two had a log-normal distribution with median Θ_0_ = (6, 6, 4.48, 7.39), (v) two uniform and two exponential distributions with median Θ_0_ = (6, 6, 6.15, 6.15), and (vi) two log-normal and two exponential distributions with median Θ_0_ = (6, 6, 6.89, 6.89). For each scenario, simulations were repeated 1000 times under 0%, 30%, and 50% censoring schemes and the results are summarized in Tables 2 to 5. We also considered the scenarios where different sites had different censoring rates (0% to 70%) in Table 6, using three mixed distributions and a sample size of 50.

**Table 2:** Mean of 1000 estimated medians and REs for 0%, 30% and 50% censoring rates for uniform, log-normal and exponential distributions when sample size is 50.

Distribution	Type	Censoring Rate = 0					
		*θ*_1_	*θ*_2_	*θ*_3_	*θ*_4_	MSE	RE
Uniform		Θ_0_ = (6, 6, 6.5, 6)					
	UE	5.968	5.958	6.460	5.956	0.312	1.000
	CE	6.086	6.086	6.086	6.086	0.295	1.058
	PP	5.992	5.984	6.384	5.980	0.261	1.194
Log-normal		Θ_0_ = (403.4, 665.1, 403.4, 665.1)					
	UE	403.7	661.5	398.0	665.0	39290	1.000
	CE	459.4	459.4	459.4	459.4	103719	0.379
	PP	411.4	636.8	405.9	640.3	37472	1.049
Exponential		Θ_0_ = (6.69, 6.58, 6.99, 6.43)					
	UE	6.680	6.566	6.984	6.432	0.049	1.000
	CE	6.639	6.639	6.639	6.639	0.206	0.238
	PP	6.678	6.570	6.964	6.444	0.048	1.027
		Censoring Rate = 0.3					
Uniform		Θ_0_ = (6, 6, 6.5, 6)					
	UE	6.012	6.002	6.511	6.007	0.414	1.000
	CE	6.118	6.118	6.118	6.118	0.325	1.273
	PP	6.036	6.032	6.419	6.031	0.327	1.265
Log-normal		Θ_0_ = (403.4, 665.1, 403.4, 665.1)					
	UE	417.0	680.3	413.7	688.6	55513	1.000
	CE	471.6	471.6	471.6	471.6	102099	0.544
	PP	427.0	647.7	423.6	654.0	49474	1.122
Exponential		Θ_0_ = (6.69, 6.58, 6.99, 6.43)					
	UE	6.701	6.590	7.003	6.449	0.063	1.000
	CE	6.651	6.651	6.651	6.651	0.213	0.298
	PP	6.699	6.595	6.977	6.464	0.060	1.050
		Censoring Rate = 0.5					
Uniform		Θ_0_ = (6, 6, 6.5, 6)					
	UE	6.024	6.019	6.525	6.018	0.496	1.000
	CE	6.122	6.122	6.122	6.122	0.346	1.432
	PP	6.050	6.048	6.425	6.042	0.381	1.300
Log-normal		Θ_0_ = (403.4, 665.1, 403.4, 665.1)					
	UE	425.1	691.1	420.4	704.4	96626	1.000
	CE	473.0	473.0	473.0	473.0	107244	0.901
	PP	438.0	643.7	433.6	655.0	77915	1.240
Exponential		Θ_0_ = (6.69, 6.58, 6.99, 6.43)					
	UE	6.708	6.593	7.015	6.457	0.091	1.000
	CE	6.649	6.649	6.649	6.649	0.227	0.400
	PP	6.703	6.600	6.972	6.478	0.083	1.091

**Table 3:** Mean of 1000 estimated medians and REs for 0%, 30% and 50% censoring rates for uniform, log-normal and exponential distributions when sample size is 100.

Distribution	Type	Censoring Rate = 0					
		*θ*_1_	*θ*_2_	*θ*_3_	*θ*_4_	MSE	RE
Uniform		Θ_0_ = (6, 6, 6.5, 6)					
	UE	5.972	5.961	6.450	5.978	0.305	1.000
	CE	6.091	6.091	6.091	6.091	0.305	1.002
	PP	5.997	5.993	6.370	6.001	0.262	1.163
Log-normal		Θ_0_ = (403.4, 665.1, 403.4, 665.1)					
	UE	399.2	662.0	401.3	656.1	39706	1.000
	CE	458.6	458.6	458.6	458.6	102924	0.386
	PP	407.5	636.1	408.7	631.6	38364	1.035
Exponential		Θ_0_ = (6.69, 6.58, 6.99, 6.43)					
	UE	6.677	6.566	6.993	6.423	0.049	1.000
	CE	6.635	6.635	6.635	6.635	0.210	0.237
	PP	6.675	6.570	6.974	6.435	0.047	1.041
		Censoring Rate = 0.3					
Uniform		Θ_0_ = (6, 6, 6.5, 6)					
	UE	6.010	6.011	6.499	6.030	0.411	1.000
	CE	6.129	6.129	6.129	6.129	0.326	1.258
	PP	6.042	6.045	6.406	6.051	0.335	1.226
Log-normal		Θ_0_ = (403.4, 665.1, 403.4, 665.1)					
	UE	414.9	681.4	415.6	679.0	55867	1.000
	CE	470.3	470.3	470.3	470.3	102184	0.547
	PP	425.9	646.4	426.2	644.6	50394	1.109
Exponential		Θ_0_ = (6.69, 6.58, 6.99, 6.43)					
	UE	6.701	6.584	7.011	6.439	0.063	1.000
	CE	6.645	6.645	6.645	6.645	0.215	0.296
	PP	6.698	6.589	6.983	6.453	0.060	1.058
		Censoring Rate = 0.5					
Uniform		Θ_0_ = (6, 6, 6.5, 6)					
	UE	6.007	6.009	6.511	6.012	0.243	1.000
	CE	6.115	6.115	6.115	6.115	0.280	0.870
	PP	6.028	6.030	6.430	6.034	0.211	1.151
Log-normal		Θ_0_ = (403.4, 665.1, 403.4, 665.1)					
	UE	413.9	679.1	408.8	675.4	34770	1.000
	CE	469.0	469.0	469.0	469.0	97229	0.358
	PP	421.1	654.4	416.7	651.1	31904	1.090
Exponential		Θ_0_ = (6.69, 6.58, 6.99, 6.43)					
	UE	6.708	6.585	7.006	6.444	0.043	1.000
	CE	6.655	6.655	6.655	6.655	0.206	0.209
	PP	6.705	6.589	6.987	6.455	0.042	1.033

**Table 4:** Mean of 1000 estimated medians and REs for 0%, 30% and 50% censoring rates for (2 uniform+2 lognormal), (2 uniform+2 exponential), (2 lognormal +2 exponential) distributions when sample size is 50.

Distribution	Type	Censoring Rate = 0					
		*θ*_1_	*θ*_2_	*θ*_3_	*θ*_4_	MSE	RE
2 uniform+2 lognormal		Θ_0_ = (6, 6, 4.48, 7.38)					
	UE	5.990	5.992	4.471	7.322	2.391	1.000
	CE	5.984	5.984	5.984	5.984	4.244	0.563
	PP	5.989	5.991	4.707	7.106	2.206	1.084
2 uniform+2 exponential		Θ_0_ = (6, 6, 6.15, 6.15)					
	UE	5.991	5.989	6.146	6.143	0.051	1.000
	CE	6.016	6.016	6.016	6.016	0.044	1.149
	PP	5.997	5.996	6.113	6.111	0.041	1.231
2 lognormal +2 exponential		Θ_0_ = (6, 6, 6.89, 6.89)					
	UE	5.953	5.978	6.887	6.881	2.228	1.000
	CE	6.845	6.845	6.845	6.845	1.473	1.512
	PP	6.140	6.160	6.879	6.875	1.645	1.354
		Censoring Rate = 0.3					
2 uniform+2 lognormal		Θ_0_ = (6, 6, 4.48, 7.38)					
	UE	6.000	6.003	4.612	7.605	3.500	1.000
	CE	5.995	5.995	5.995	5.995	4.247	0.824
	PP	6.000	6.002	4.881	7.284	2.964	1.181
2 uniform+2 exponential		Θ_0_ = (6, 6, 6.15, 6.15)					
	UE	6.000	5.999	6.172	6.167	0.067	1.000
	CE	6.025	6.025	6.025	6.025	0.042	1.575
	PP	6.006	6.006	6.133	6.128	0.049	1.359
2 lognormal +2 exponential		Θ_0_ = (6, 6, 6.89, 6.89)					
	UE	6.134	6.192	6.912	6.904	3.447	1.000
	CE	6.864	6.864	6.864	6.864	1.544	2.232
	PP	6.313	6.353	6.901	6.896	2.311	1.492
		Censoring Rate = 0.5					
2 uniform+2 lognormal		Θ_0_ = (6, 6, 4.48, 7.38)					
	UE	6.004	6.007	4.666	7.722	5.253	1.000
	CE	5.995	5.995	5.995	5.995	4.251	1.236
	PP	6.003	6.005	4.981	7.282	4.115	1.277
2 uniform+2 exponential		Θ_0_ = (6, 6, 6.15, 6.15)					
	UE	6.003	6.003	6.184	6.177	0.099	1.000
	CE	6.023	6.023	6.023	6.023	0.047	2.124
	PP	6.009	6.011	6.133	6.125	0.068	1.448
2 lognormal +2 exponential		Θ_0_ = (6, 6, 6.89, 6.89)					
	UE	6.221	6.289	6.924	6.910	5.024	1.000
	CE	6.858	6.858	6.858	6.858	1.542	3.259
	PP	6.396	6.447	6.906	6.898	3.102	1.620

**Table 5:** Mean of 1000 estimated medians and REs for 0%, 30% and 50% censoring rates for (2 uniform+2 lognormal), (2 uniform+2 exponential), (2 lognormal +2 exponential) distributions when sample size is 100.

Distribution	Type	Censoring Rate = 0					
		*θ*_1_	*θ*_2_	*θ*_3_	*θ*_4_	MSE	RE
2 uniform+2 lognormal		Θ_0_ = (6, 6, 4.48, 7.38)					
	UE	5.989	5.988	4.413	7.361	2.216	1.000
	CE	5.982	5.982	5.982	5.982	4.242	0.522
	PP	5.988	5.987	4.637	7.122	2.019	1.097
2 uniform+2 exponential		Θ_0_ = (6, 6, 6.15, 6.15)					
	UE	5.989	5.993	6.144	6.141	0.050	1.000
	CE	6.017	6.017	6.017	6.017	0.045	1.112
	PP	5.997	5.999	6.112	6.108	0.041	1.226
2 lognormal +2 exponential		Θ_0_ = (6, 6, 6.89, 6.89)					
	UE	6.029	5.952	6.884	6.867	2.363	1.000
	CE	6.839	6.839	6.839	6.839	1.455	1.625
	PP	6.183	6.148	6.876	6.862	1.687	1.401
		Censoring Rate = 0.3					
2 uniform+2 lognormal		Θ_0_ = (6, 6, 4.48, 7.38)					
	UE	6.001	5.999	4.549	7.552	3.104	1.000
	CE	5.994	5.994	5.994	5.994	0.246	0.731
	PP	5.999	5.998	4.826	7.206	2.629	1.180
2 uniform+2 exponential		Θ_0_ = (6, 6, 6.15, 6.15)					
	UE	5.999	6.004	6.172	6.168	0.064	1.000
	CE	6.024	6.024	6.024	6.024	0.043	1.480
	PP	6.006	6.010	6.131	6.126	0.048	1.334
2 lognormal +2 exponential		Θ_0_ = (6, 6, 6.89, 6.89)					
	UE	6.217	6.096	6.908	6.888	3.274	1.000
	CE	6.856	6.856	6.856	6.856	1.516	2.159
	PP	6.367	6.288	6.896	6.880	2.096	1.562
		Censoring Rate = 0.5					
2 uniform+2 lognormal		Θ_0_ = (6, 6, 4.48, 7.38)					
	UE	6.001	6.003	4.515	7.542	2.151	1.000
	CE	5.994	5.994	5.994	5.994	4.241	0.507
	PP	6.001	6.002	4.735	7.290	1.932	1.114
2 uniform+2 exponential		Θ_0_ = (6, 6, 6.15, 6.15)					
	UE	6.002	6.002	6.158	6.164	0.042	1.000
	CE	6.024	6.024	6.024	6.024	0.041	1.025
	PP	6.007	6.008	6.124	6.130	0.033	1.266
2 lognormal +2 exponential		Θ_0_ = (6, 6, 6.89, 6.89)					
	UE	6.081	6.146	6.908	6.907	2.126	1.000
	CE	6.869	6.869	6.869	6.869	1.544	1.377
	PP	6.279	6.321	6.898	6.899	1.576	1.349

**Table 6:** Mean of 1000 estimated medians and REs when censoring rates are different among 4 sites (small censoring rates: 30%, 20%, 10%, 0%; large censoring rates: 70%, 50%, 30%, 10%), for (2 uniform+2 lognormal), (2 uniform+2 exponential), (2 lognormal +2 exponential) distributions when sample size is 50.

Distribution	Type	Censoring Rate = (30%, 20%, 10%, 0%)					
		*θ*_1_	*θ*_2_	*θ*_3_	*θ*_4_	MSE	RE
2 uniform+2 lognormal		Θ_0_ = (6, 6, 4.48, 7.38)					
	UE	6.000	6.004	4.588	7.322	2.518	1.000
	CE	5.995	5.995	5.995	5.995	4.246	0.593
	PP	5.999	6.003	4.836	7.086	2.331	1.080
2 uniform+2 exponential		Θ_0_ = (6, 6, 6.15, 6.15)					
	UE	5.999	6.000	6.168	6.143	0.056	1.000
	CE	6.027	6.027	6.027	6.027	0.041	1.363
	PP	6.006	6.007	6.133	6.113	0.043	1.281
2 lognormal +2 exponential		Θ_0_ = (6, 6, 6.89, 6.89)					
	UE	6.134	6.151	6.907	6.881	3.090	1.000
	CE	6.859	6.859	6.859	6.859	1.521	2.032
	PP	6.307	6.315	6.896	6.877	2.117	1.460
		Censoring Rate = (70%, 50%, 30%, 10%)					
2 uniform+2 lognormal		Θ_0_ = (6, 6, 4.48, 7.38)					
	UE	6.005	6.004	4.612	7.514	2.854	1.000
	CE	5.996	5.996	5.996	5.996	4.254	0.671
	PP	6.005	6.003	4.875	7.219	2.531	1.127
2 uniform+2 exponential		Θ_0_ = (6, 6, 6.15, 6.15)					
	UE	6.002	6.001	6.172	6.161	0.068	1.000
	CE	6.035	6.035	6.035	6.035	0.042	1.611
	PP	6.011	6.011	6.136	6.127	0.051	1.340
2 lognormal +2 exponential		Θ_0_ = (6, 6, 6.89, 6.89)					
	UE	6.180	6.288	6.899	6.880	5.597	1.000
	CE	6.847	6.847	6.847	6.847	1.531	3.656
	PP	6.354	6.409	6.888	6.874	3.701	1.512

In general, the results in Tables 2 and 6 show that survival distribution and censoring rate affect the deviation from the true value for different estimators. The RE of ${\hat{\Theta }}^{CE}$ ranges from 20% to over 300%, which might be considered as an approximate measure of departure from the homogeneity of survival medians. Overall, shrinkage estimator ${\hat{\Theta }}^{PP}$ outperforms ${\hat{\Theta }}^{CE}$ with respect to UE in all aforementioned scenarios, yielding 100% to 160% REs. Efficiency gain is more noticeable with higher censoring rate.

## Application to the PROMMTT study

The PROMMTT study was a multi-site prospective observational cohort study in a severely injured transfused trauma patients, conducted at 10 level 1 trauma centers in the United States [1, 11]. The original objectives of PROMMTT study were to accurately describe when some blood components were infused and to assess the association between in-hospital mortality and the timing and amount of blood products. Understanding current blood product usage patterns and their impact on patient outcomes among a severely injured and substantially hemorrhaging cohort is critically important.

In our analysis, we applied our method on 698 patients from four major medical centers to examine difference of their median times of receiving the first unit of RBC infusion. The number of patients in the 4 sites are 303, 137, 133, and 125. The median age of patients are 34, 37, 34, and 41 years. The percents of male patients are 73.27%, 77.37%, 76.69%, and 75.20%. Around 61.06%, 84.67%, 54.26%, and 96.77% are Caucasian in the four sites, respectively. Since no patients dropped out of study before the first unit of RBC infusion, the censoring rate for each site is zero.

**Fig 2:**
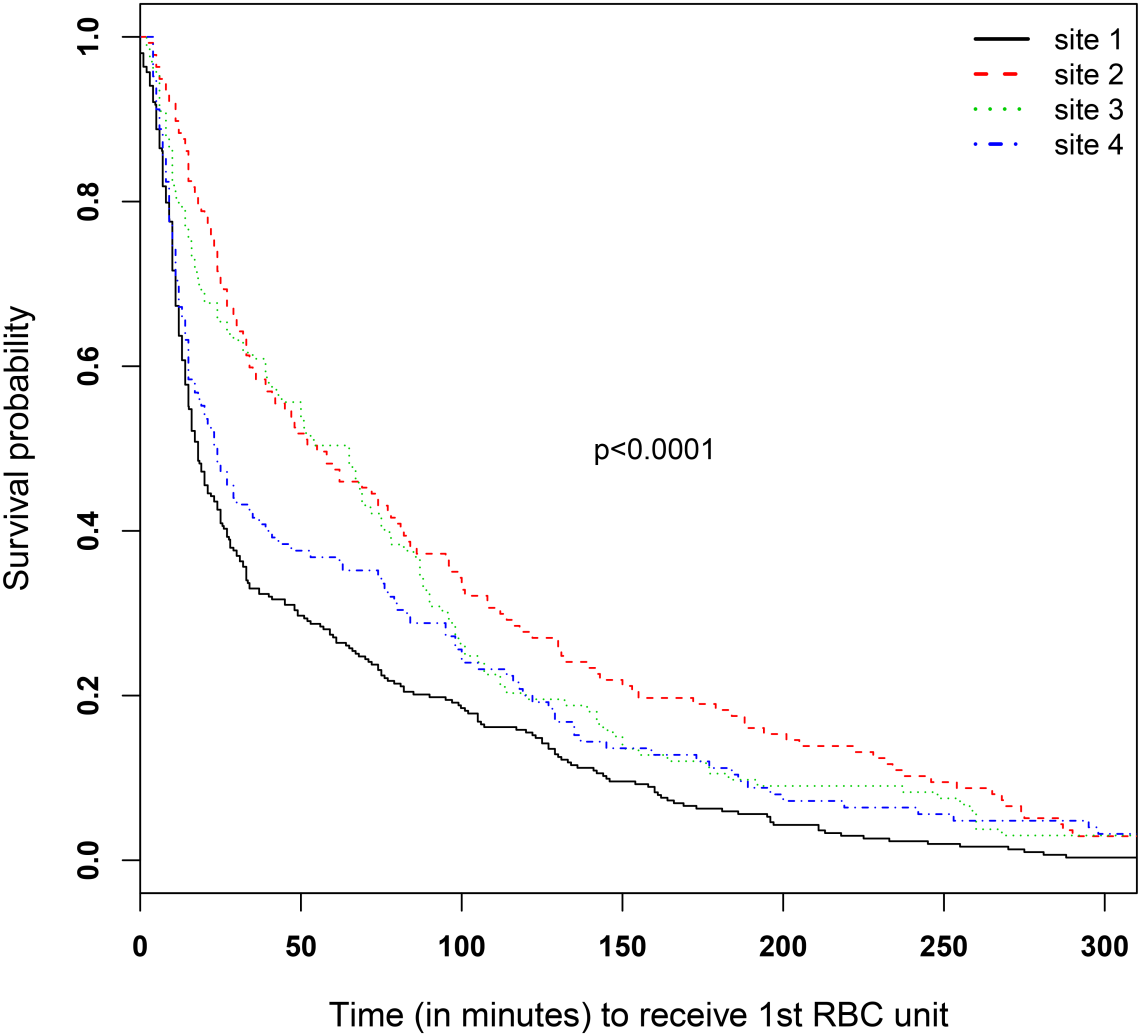
The survival curves of the four Kaplan-Meier (KM) estimates for time to receiving the first unit of RBC from site 1 (n1 = 303, solid line), site 2 (n2 = 137, dashed), site 3 (n3 = 133, dotted), and site 4 (n4 = 125, dot-dashed), respectively.

We apply the proposed method to this data in two steps. First, we formally evaluate homogeneity of medians using randomly right censored data. Second, based on the results of the test of homogeneity our proposed method evaluates whether the unrestricted estimator (UE), combined estimator (CE), or the Positive Part shrinkage estimator (PP) should be used to obtain a more efficient estimator. The primary outcome is time to the first unit of RBC infusion measured as the number of minutes from ED admission. This outcome may reflect patient’s status and may differ across different medical institutions because management of severely hemorrhaging patients may differ. As we found in the data, the UE median times to the first unit of RBC infusion are 18, 55, 65, and 24 for four sites, indicating potential heterogeneity of survival times across 4 sites. Fig 2 shows the estimated survival curves of time to the first unit of RBCs for each site, along with p-values from (2.2) and the log-rank test. It shows that median times are significantly different, suggesting that caution should be exercised when merging data sets from different sites. All of these motivate us to apply the proposed Positive Part shrinkage estimator (PP) method.

Table 7 summarizes estimated median times and their 95% confidence interval based on bootstrap variance for each site based on PP method, along with the comparisons to CE and UE methods. The PP median times are 18.08, 54, 63.71, and 23.91, compared to the CE median times of 20.82, 20.82, 20.82, and 20.82. Obviously, the CE median times are unreliable since the test for homogeneity of medians to first RBC infusion is rejected (p-value<0.0001). The PP median times are almost identical to the UE median times, with slightly narrower confidence intervals than UE median times at site 2, 3, and 4, indicating a slight efficiency gain.

**Table 7:** Estimated median time to receive the first unit of red blood cell (RBC) infusion in PROMMTT study by study sites.

Type of Estimator	Median time to receive the first unit of RBC Infusion (in minutes)			
	site 1 (*n*_1_ = 303)	site 2 (*n*_2_ = 137)	site 3 (*n*_3_ = 133)	site 4 (*n*_4_ = 125)
UE	18 (15 - 21)	55 (39 - 80)	65 (39 - 75)	24 (15 - 37)
CE	20.8 (16.7 - 27.4)	20.8 (16.7 - 27.4)	20.8 (16.7 - 27.4)	20.8 (16.7 - 27.4)
PP	18.1 (15.0 - 21.3)	54 (37.7 - 77.4)	63.7 (38.3 - 73.9)	23.9 (15.2 - 36.5)

## Concluding remarks

In this paper, other than the unrestricted estimator and simply combined estimator, we have presented a shrinkage nonparametric approach for estimating the median survival vector in a *k*-sample problem. Other than asymptotical comparison on ADQR, extensive simulations have been done to assess the performance of these estimators, considering various scenarios allowing varying levels of censorship and different level of departure from homogeneity of the survival medians.

The performance of the combined estimator heavily depends on the strength of homogeneity. When homogeneity holds ${\hat{\Theta }}^{CE}$ is more efficient compared to ${\hat{\Theta }}^{UE}$ and ${\hat{\Theta }}^{PP}$. However, ${\hat{\Theta }}^{CE}$ becomes inconsistent and the efficiency of the CE decreases significantly when homogeneity fails. On the other hand, ${\hat{\Theta }}^{PP}$ seems to be robust to the non-homogeneity and different levels of censoring. Though the relative efficiency against ${\hat{\Theta }}^{UE}$ decreases as we deviate from the quality of the survival medians, it keeps being greater or equal to 1. Like any other shrinkage estimation procedures, there is a bias-variance tradeoff for ${\hat{\Theta }}^{PP}$.

The proposed procedures have applications in epidemiologic and health care research. For example, for estimating survival median based on data from a multi-site study one always faces with the challenge of whether to pool data from all sites or not pool such data. In this study using PROMMTT data we have demonstrated the utility of various estimators as well as how one can make a decision as to choose the most appropriate estimation procedure. On the other hand, if the distributions of median times are similar, greater efficiency of the estimators by using shrinkage-type methods may be gained, depending on the distribution of event time.

## Supporting information

S1 File Revised PROMMTT publication committee guidelines. (DOCX)

S1 Dataset R code for generating simulated data and analysis. (ZIP)
