# Supplementary material for: Nonparametric estimation of median survival times with applications to multi-site or multi-center studies
Source: PLoS One. 2018 May 17;13(5):e0197295. doi: 10.1371/journal.pone.0197295 (PMC5957417; doi:10.1371/journal.pone.0197295)
Supplement: S1 File — (DOCX) [file pone.0197295.s001.docx]

**
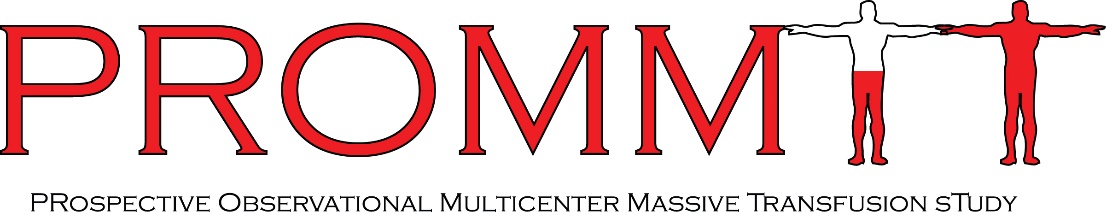
**

**PROMMTT**

**Publication Committee Guidelines**

The following guidelines have been established by the PROMMTT Publication Committee for distribution of data maintained by the Data Coordinating Center. These guidelines refer to multi-center data only and not site specific data.

**[A*ll data were collected under the protocol titled: PRospective Observational Multicenter Major Trauma Transfusion sTudy]***

Cleaned and locked data sets will be sent to all PROMMTT site PIs.

- The dataset is not to be shared with individuals who are not part of the PROMMTT study group.
- All abstracts, manuscripts and other potentially publishable products will be reviewed by the publication committee. The publication committee will review all submissions to ensure absence of conflict with previously approved projects, determine merit, and record date and approval of the submission. There will be a member from each site and the DCC on the Publication Committee. Members of the publication committee will also have the opportunity to voluntarily assist with the proposed research and form a writing group.
- Any extrinsic use of PROMMTT dataset must be approved by the publication committee.

The following guidelines have been established by the PROMMTT Publication Committee for submitting a manuscript to be screened:

- A manuscript review request form must be submitted along with a copy of the manuscript to the publication committee. A manuscript review request can be obtained by contacting [PROMMTT@UTH.TMC.EDU](mailto:PROMMTT@UTH.TMC.EDU)
- Committee will briefly screen manuscript for quality.
- Authorship: The person who proposes the project and performs the majority of the work will be the 1^st^ author. Additional authors must meet the requirements for authorship as described by the Journal of the American Medical Association. Manuscripts with authors from multiple institutions will be limited to 2 authors per study site and the DCC.
- The DCC faculty/staff are permitted to utilize the multisite data for their own methodological research.  Methodological research includes development of statistical techniques but in order to publish such results in applied statistical journals there is a need to show that the new proposed statistical methodology works in a real life situation.  These requests will be subject to the same guidelines that are outlined in this document.
- Access to data will be limited to PROMMTT investigators for a period of 2 years following publication of the first manuscript. Following this period, outside investigators will be able to submit requests for data. These requests will subject to the same guidelines that are outlined in this document.
- Each site is permitted to utilize their own data for analysis and publication after it is released to them by the Data Coordination Center.
- Our study group will be known as The PROMMTT Study Group.
